# Supplementary material for: The causal role of circulating inflammatory markers in osteoporosis: a bidirectional Mendelian randomized study
Source: Front Immunol. 2024 Jul 18;15:1412298. doi: 10.3389/fimmu.2024.1412298 (PMC11291241; doi:10.3389/fimmu.2024.1412298)
Supplement: Supplementary file 1 [file DataSheet_1.zip › supplementary material/OSTEOPOROSIS-CIM/GCST90274773.tsv.gz_leave_one_out.pdf]

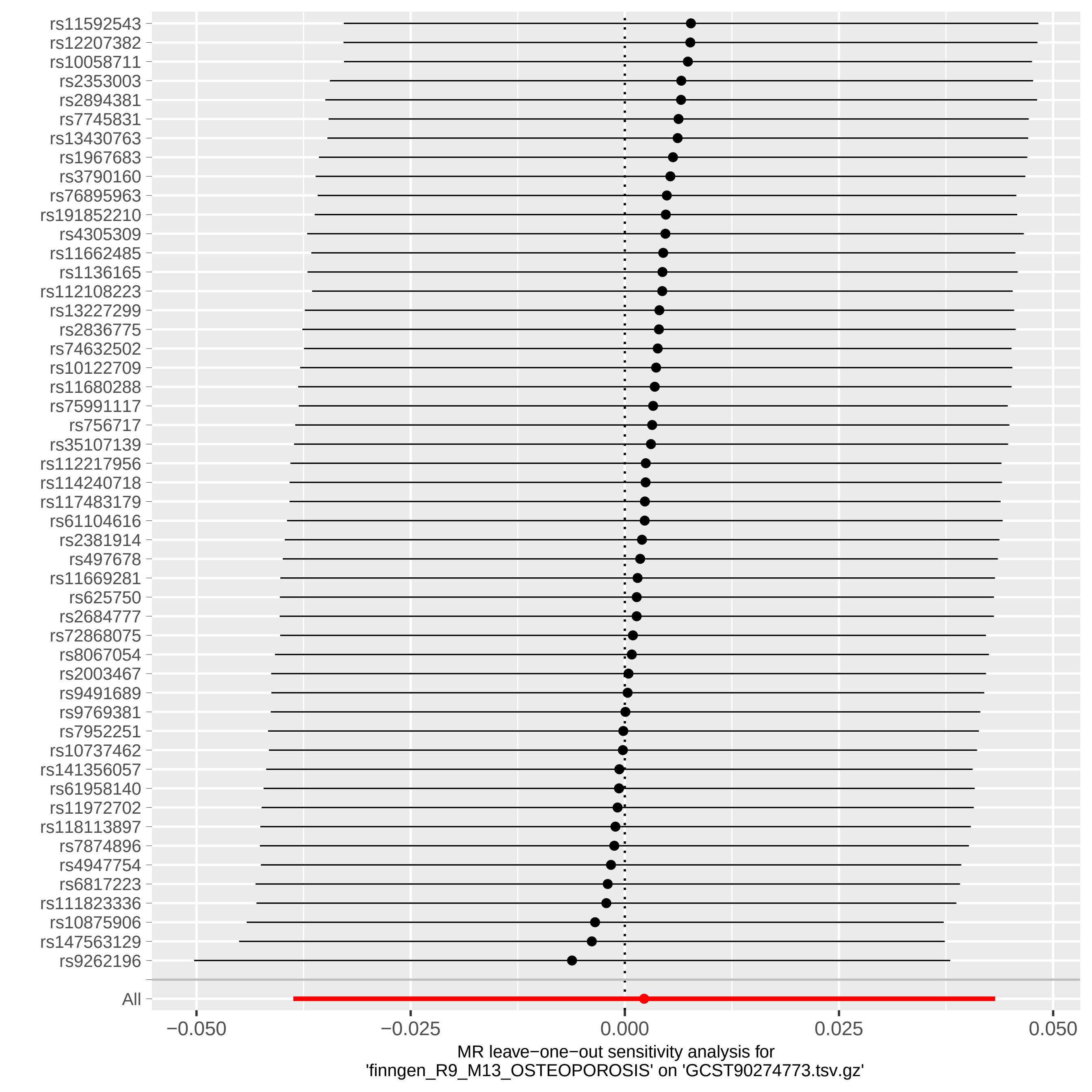
rs11592543
rs12207382
rs10058711
rs2353003
rs2894381
rs7745831
rs13430763
rs1967683
rs3790160
rs76895963
rs191852210
rs4305309
rs11662485
rs1136165
rs112108223
rs13227299
rs2836775
rs74632502
rs10122709
rs11680288
rs75991117
rs756717
rs35107139
rs112217956
rs114240718
rs117483179
rs61104616
rs2381914
rs497678
rs11669281
rs625750
rs2684777
rs72868075
rs8067054
rs2003467
rs9491689
rs9769381
rs7952251
rs10737462
rs141356057
rs61958140
rs11972702
rs118113897
rs7874896
rs4947754
rs6817223
rs111823336
rs10875906
rs147563129
rs9262196
All
−0.050
−0.025
0.000
0.025
0.050
MR leave−one−out sensitivity analysis for
'finngen_R9_M13_OSTEOPOROSIS' on 'GCST90274773.tsv.gz'
